# Causal association between depression and intracranial aneurysms: a bidirectional two-sample Mendelian randomization study

**DOI:** 10.3389/fneur.2024.1346703

**Published:** 2024-02-14

**Authors:** Jujiang Wu, Hao Sun, Junqiang Ma

**Affiliations:** ^1^Neurointensive Care Unit, The First Affiliated Hospital of Shantou University Medical College, Shantou, China; ^2^Department of Population Medicine, Shantou University Medical College, Shantou, China

**Keywords:** causal association, depression, intracranial aneurysms, subarachnoid hemorrhage, Mendelian randomization

## Abstract

**Background:**

Although observational studies have suggested a bidirectional relation between depression and intracranial aneurysms (IAs), their causal relations remain unclear. Thus we aimed to assess the causal association between depression and IAs.

**Methods:**

We conducted a bidirectional two-sample Mendelian randomization (MR) study using summary-level data from publicly available genome-wide association studies of depression (*n* = 500,199), IAs (*n* = 79,429), unruptured intracranial aneurysm (uIA) (*n* = 74,004), and subarachnoid hemorrhage (SAH) (*n* = 77,074). MR analyses included the inverse-variance weighted (IVW) method as the primary analytic, plus weighted-median, simple mode, weighted mode, MR-Egger, and MR PRESSO.

**Results:**

Genetically predicted depression was strongly positively related to IAs (odds ratio [OR] = 1.69, 95% confidence interval [CI] 1.19–2.39, *p* = 0.003), uIA (OR = 1.96, 95% CI 1.06–3.64, *p* = 0.032), and SAH (OR = 1.73, 95% CI 1.14–2.61, *p* = 0.009). Reverse MR analyses showed that while genetically predicted uIA was positively related to depression (OR = 1.02, 95% CI 1.00–1.05, *p* = 0.044), no causal relations were observed for either IAs or SAH for depression.

**Conclusion:**

Our findings provide evidence of a causal effect of depression on IAs, uIA, and SAH. For the reverse MR analyses, we found a causal impact of uIA on depression, but no causal influence of either IAs or SAH for depression.

## 1 Introduction

Intracranial aneurysm (IA), localized pathological dilations at major bifurcations of cerebral arteries, is characterized by internal elastic lamina loss and media disruption (1). In a global study of individuals with a mean age of 50 years, IA incidence was ~3.2% (2). IAs are susceptible to rupture, which causes ~85% of spontaneous subarachnoid hemorrhage (SAH) (3). Poor prognosis and high death and disability rates are common features of aneurysmal SAH (4, 5). Consequently, it is important to pinpoint the causes of IA development to launch early, targeted interventions.

Depression is a primary cause of disability, accounting for over 300 million cases globally (6). Those with depression are at increased risk for a host of medical conditions in later life (7). Observational studies have shown a connection between IA and depression (8–10). For instance, a nine-year cohort study by Marijnissen et al. revealed depression to be a stroke (including IA) risk factor (11). However, as has been frequently noted, these observational studies have been limited by confounding factors and reverse causality. Because development of both depression and IA have vague, subtle onsets, it is challenging to establish their temporal order. One study revealed a lack of genetic support for a causal relation between major depressive disorder and IA (12), possibly because of confounding factors and differences in data sources. There has also been insufficient evidence to determine the direction of such causality (12); thus, the potential causality of depression in IA risk, and vice versa, has remained elusive.

Mendelian randomization (MR), which uses genetic variation in non-experimental data to identify causal relations between exposure and outcome, can lessen the statistical influence of social, behavioral, psychological, and other factors (13). By utilizing genome-wide association study (GWAS) summary statistics, MR studies have emerged as a powerful, effective tool for determining causal relations between exposure and outcome phenotypes (14, 15). Using single nucleotide polymorphisms (SNPs) extracted as instrumental variables (IVs) from a GWAS, a two-sample MR analysis can be used to determine causal links between two traits (16). Herein, we analyzed the causal relations between depression and IAs with two-sample MR with recently published GWAS summary data for depression and IAs.

## 2 Methods

**Figure 1:**
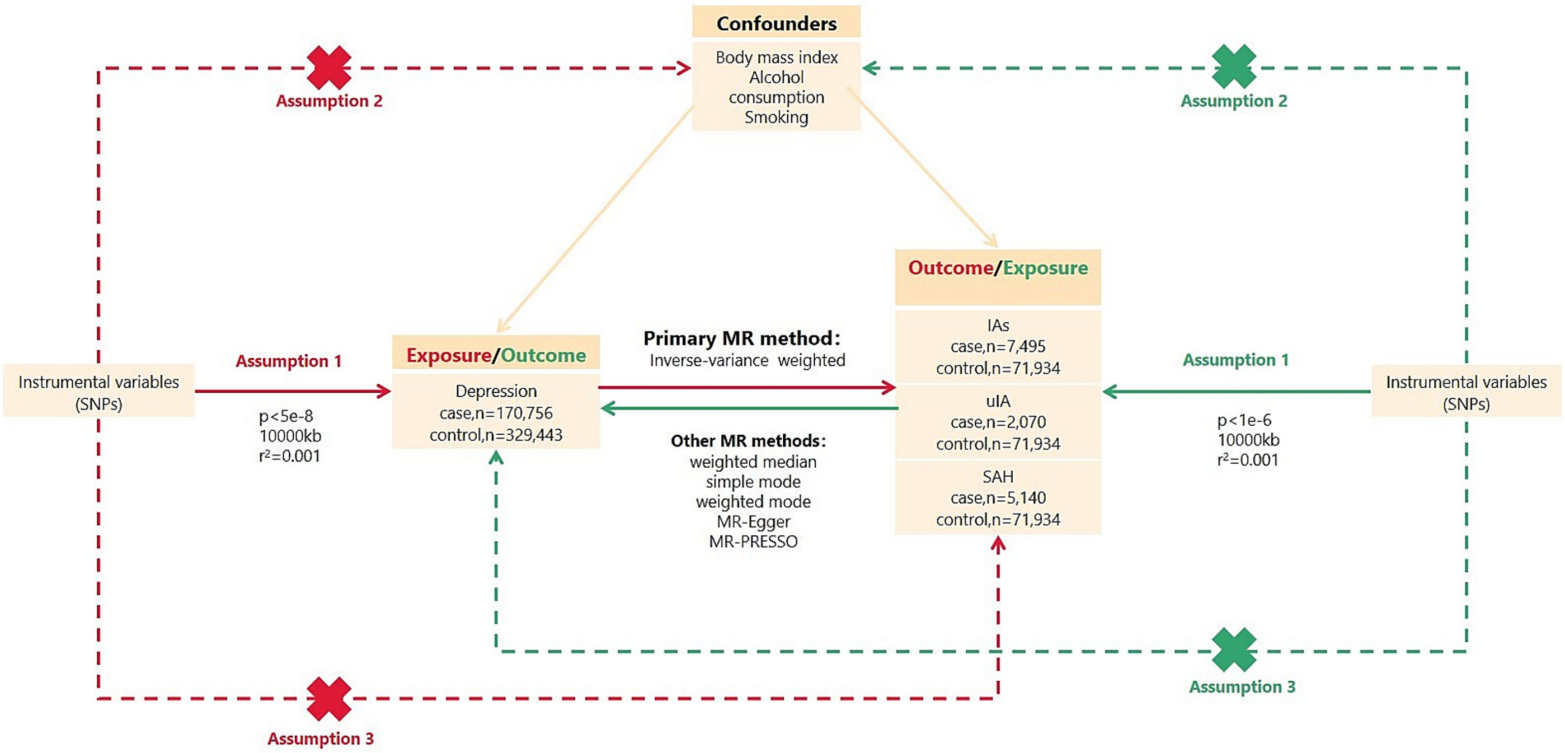
Overview of bidirectional MR study design. SNPs, single nucleotide polymorphisms; IVs, instrumental variables; IAs, intracranial aneurysms; uIA, unruptured intracranial aneurysm; SAH, subarachnoid hemorrhage; MR, Mendelian randomization. MR analysis depends on three major assumptions: Assumption 1, IVs are strongly associated with exposure; Assumption 2, IVs are independent of confounders; Assumption 3, IVs are not directly related to outcomes.

To assess the causal association between depression and IAs, we performed a bidirectional two-sample MR analysis for each exposure–outcome pair. All GWAS summary data analyzed herein are publicaly available. All included studies obtained ethical approval and informed consent. Figure 1 shows a brief description of this bidirectional MR design. Summary GWAS data for depression and IAs were assembled from published studies using samples from the most significant European populations (Table 1).

**Table 1:** GWAS data details.

Phenotype	Total sample size	Cases, *n*	Controls, *n*	Population	Consortium	PMID
Depression	500,199	170,756	329,443	European	Howard et al.	30718901
IAs	79,429	7,495	71,934	European	Bakker et al.	33199917
uIA	74,004	2,070	71,934	European	Bakker et al.	33199917
SAH	77,074	5,140	71,934	European	Bakker et al.	33199917

IAs, intracranial aneurysms; uIA, unruptured intracranial aneurysm; SAH, subarachnoid hemorrhage.

### 2.1 Genetic instrument selection for MR analyses

The three major MR assumptions were used to filter the SNPs for each exposure factor. For assumption 1, we performed the following three steps. First, SNPs that met a threshold for genome-wide significance (*p* < 5 × 10^−8^) and were associated with the exposure were included as IVs. Second, based on linkage disequilibrium (LD) as determined by *r*^2^ and window size (when *r*^2^ < 0.001 and window size = 10,000 kb in the European 1,000 Genome reference panel), we retained variations with the lowest *p*-values as independent instruments. Third, we calculated F-statistics to quantify the power of IVs; F-statistics >10 is commonly advised for MR analysis. For assumption 2, the IVs did not include SNPs that were significantly (*p* < 1 × 10^−5^) linked to confounders. We controlled for confounding factors, including body mass index, alcohol intake frequency, and smoking (Supplementary Table S1). For assumption 3, SNPs related to outcomes were eliminated from the IVs.

### 2.2 Depression data

The three largest GWAS including various depression phenotypes from 23andMe, PGC, and UK Biobank were included in the depression meta-analysis by Howard et al. (17). However, in only PGC and UK Biobank are the meta-analyses of depression summary statistics for all assessed variants publicly available; these are based on 170,756 cases and 329,443 controls, all of whom are of European ancestry. Therefore, the primary analysis herein examined the association between depression and IAs based on 500,199 individuals with depression, as defined by the PGC and UK Biobank meta-analyses. Using the *p* < 5 × 10^−8^ threshold, 20 SNPs were found to be associated with depression for IVs (Supplementary Table S2). The reverse-direction MR analyses also used the summary-level PGC and UK Biobank data for 500,199 individuals.

### 2.3 IAs, uIA and SAH data

Summary statistics IAs data among individuals of European ancestry originated from a GWAS of 23 different cohorts, comprised of 7,495 cases and 71,934 controls (18). Within these, 4,471,083 SNPs met the quality control standards. Among these, there were 69% with ruptured IAs, 28% with unruptured IA (uIA), and 3.8% with unknown rupture status; specifically, there were 5,140 SAH (i.e., ruptured IA) cases and 2,070 unruptured IAs cases of European ancestry. Therefore, the MR analyses herein used the three summary datasets, all including individuals of European ancestry, separately: GWAS of IAs (unruptured and ruptured) cases (*n* = 7,495) vs. controls (*n* = 71,934); GWAS of unruptured IA-only cases (*n* = 2,070) vs. controls (*n* = 71,934); and GWAS of SAH-only cases (*n* = 5,140) vs. controls (*n* = 71,934). For reverse MR analyses, since few SNP–IA associations met the genome-wide association threshold (i.e., *p* < 5 × 10^−8^), a suggestive level of significance (*p* < 1 × 10^−6^) was used to extract IVs. Eight independent genetic SNPs associated with IAs (Supplementary Table S3), 4 SNPs associated with uIA (Supplementary Table S4), and 8 SNPs associated with SAH (Supplementary Table S5) were identified at this genome-wide significance level.

### 2.4 MR analysis

Herein, *R*^2^ was the proportion of variance in an exposure factor explained by each IV, and the F-statistic was calculated to measure the strength of each IV (19). The F-statistic used the following formula: *F* = *R*^2^ (*N* − 2) / (1 − *R*^2^), where *N* = the GWAS sample size for the exposure association.

MR analyses used the random-effects inverse-variance weighted (IVW) method as the primary analysis to assess the potential bidirectional causal relations between depression and IAs, because it gives a reliable causal estimate in the absence of directional pleiotropy (20). We also performed sensitivity analyses using weighted median, simple mode, weighted mode, MR-Egger, and MR-PRESSO. These robust analytics provide valid causal inferences under weaker assumptions than does the standard IVW (21, 22). The intercept of MR-Egger (23) and the global test from MR-PRESSO (24) were used to assess horizontal pleiotropy. To detect and correct horizontal pleiotropic outliers, we also used the MR pleiotropy residual sum and outlier (MR-PRESSO) approach (24). We assessed potential heterogeneity with Cochran’s *Q* (25). Leave-one-out analysis was conducted by sequentially excluding each SNP, and an IVW approach was applied to the remaining SNPs to determine whether a specific variant would impact the estimations. MR results are presented as odds ratios (OR) of the outcome risk for the corresponding unit changes in exposure, and 95% confidence intervals (CI).

All statistical analyses were performed using R (v4.3.1) statistical software. R package TwoSampleMR was used to perform MR analyses.

## 3 Results

### 3.1 Causal effects of depression on IAs, uIA, and SAH

**Figure 2:**
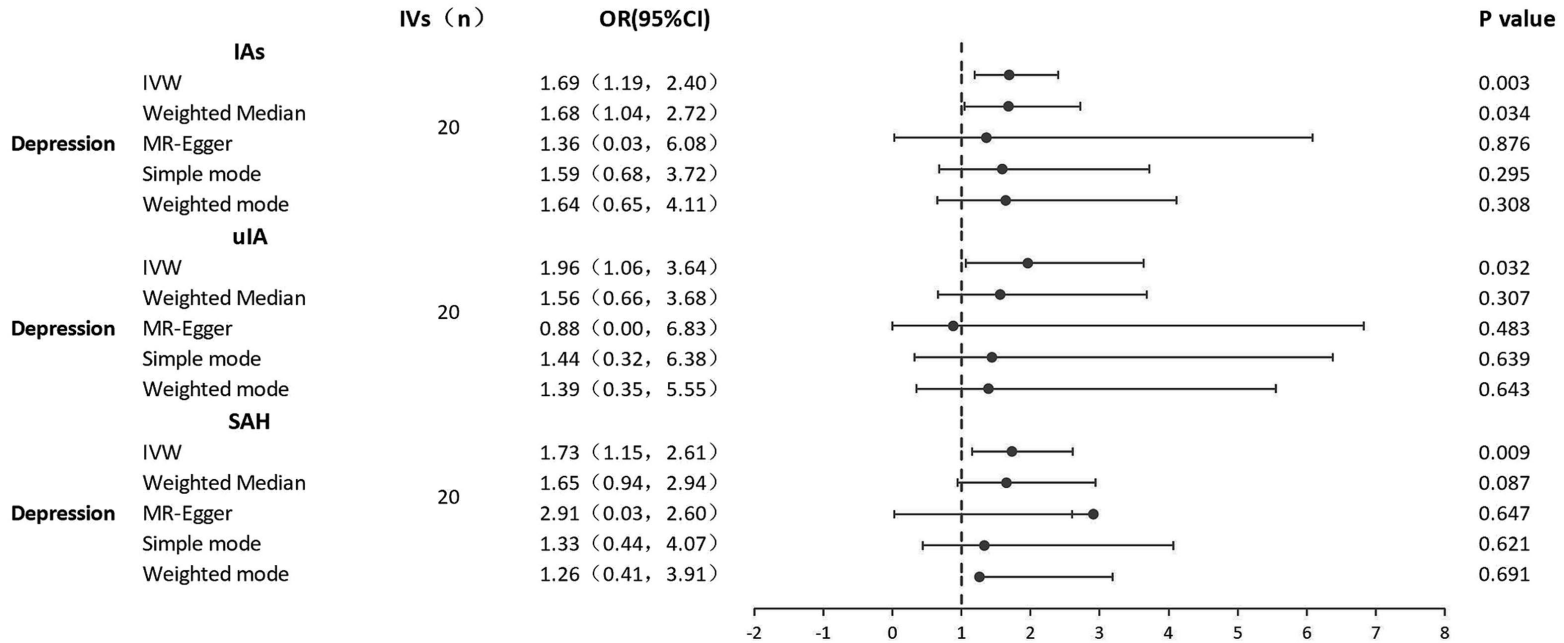
Forest plots of causal effects of depression on IAs, uIA, and SAH. IAs, intracranial aneurysms; uIA, unruptured intracranial aneurysm; SAH, subarachnoid hemorrhage; IVs, instrumental variables; IVW, inverse-variance weighted; MR, Mendelian randomization; OR, odds ratio; 95% CI, 95% confidence interval.

MR analyses of the causal effect of depression on IAs, uIA, and SAH, and pleiotropy effect assessments, are presented in Table 2. Supplementary Figures S1A–C shows scatter plots of the causal connections between depression and IAs, uIA, and SAH, with colored lines denoting the slopes of each regression analysis. In the scatter plots, each point represents a IV SNP, and lines of different colors represent different MR analysis methods. Results shown in the three scatter plots indicate that the lines representing different MR analysis methods generally slope upwards, suggesting that as depression increases, risk of developing IAs, uIA, and SAH increases. Forest plots (Figure 2) show MR estimates for the effects of the SNPs related to depression on IAs, uIA, and SAH. In the forest plots, each solid horizontal line represents a single SNP; because of the lack of robustness of the individual SNP results, it was necessary to integrate them (combined red line at bottom). The red lines below the three forest plots indicate that an increase in depression can increase the risk of IAs, uIA, and SAH.

**Table 2:** Effect estimates of associations between genetic instrumental variables for depression and aneurysm risk.

Depression	IVW		MR-Egger		MR PRESSO (outlier-corrected)		
	OR (95% CI)	Q (*p* value)	OR (95% CI)	Intercept (*p* value)	Outlier	OR (95%CI)	*p* for global test
IAs	1.69 (1.19, 2.39)	12.5 (0.86)	1.36 (0.03, 6.08)	0.0 (0.91)	0	NA	0.88
SAH	1.73 (1.14, 2.61)	13.9 (0.79)	2.91 (0.03, 2.60)	0.0 (0.79)	0	NA	0.82
uIA	1.96 (1.06, 3.64)	15.0.5 (0.69)	0.08 (0.0, 6.83)	0.09 (0.69)	0	NA	0.70

IAs, intracranial aneurysms; uIA, unruptured intracranial aneurysm; SAH, subarachnoid hemorrhage; OR, odds ratio; 95% CI, 95% confidence interval; Q, Cochran’s Q; NA, not applicable.

There were no weak IVs, as each F-statistic was not <30. Genetically predicted depression was strongly positively related to IAs, uIA, and SAH. With 20 genetic instruments in the major analysis, the IVW method demonstrated that genetically doubling the odds of depression increased the risk of IAs, SAH, and uIA by 69, 73, and 96%, respectively (IAs: OR = 1.69, 95% CI 1.19–2.39, *p* = 0.003; SAH: OR = 1.73, 95% CI 1.14–2.61, *p* = 0.009; uIA: OR = 1.96, 95% CI 1.06–3.64, *p* = 0.032). MR-Egger regression showed no horizontal pleiotropy in the analysis of the causal effect of depression on IAs, SAH or uIA (IAs: egger_intercept = 0.0, *p* = 0.91; SAH: egger_intercept = 0.0, *p* = 0.79; uIA: egger_intercept = 0.09, *p* = 0.69). Nor was horizontal pleiotropy found in the MR-PRESSO global test (IAs: *p* = 0.88; SAH: *p* = 0.82; uIA: *p* = 0.70) and MR-PRESSO failed to find any notable outliers. Cochran’s *Q* indicated no significant heterogeneities (IAs: Q = 12.5, *p* = 0.86; SAH: Q = 13.9, *p* = 0.79; uIA: Q = 15.5, *p* = 0.69); this is shown in the funnel plots (Supplementary Figures S2A–C), on which points on either side of the IVW line are roughly symmetrical. The leave-one-out test revealed that the causal estimate was not driven by any single SNP (Supplementary Figures S3A–C); that the overall error line does not change significantly after excluding each SNP indicates reliable results.

### 3.2 Causal effects of IAs, SAH, and uIA on depression

**Figure 3:**
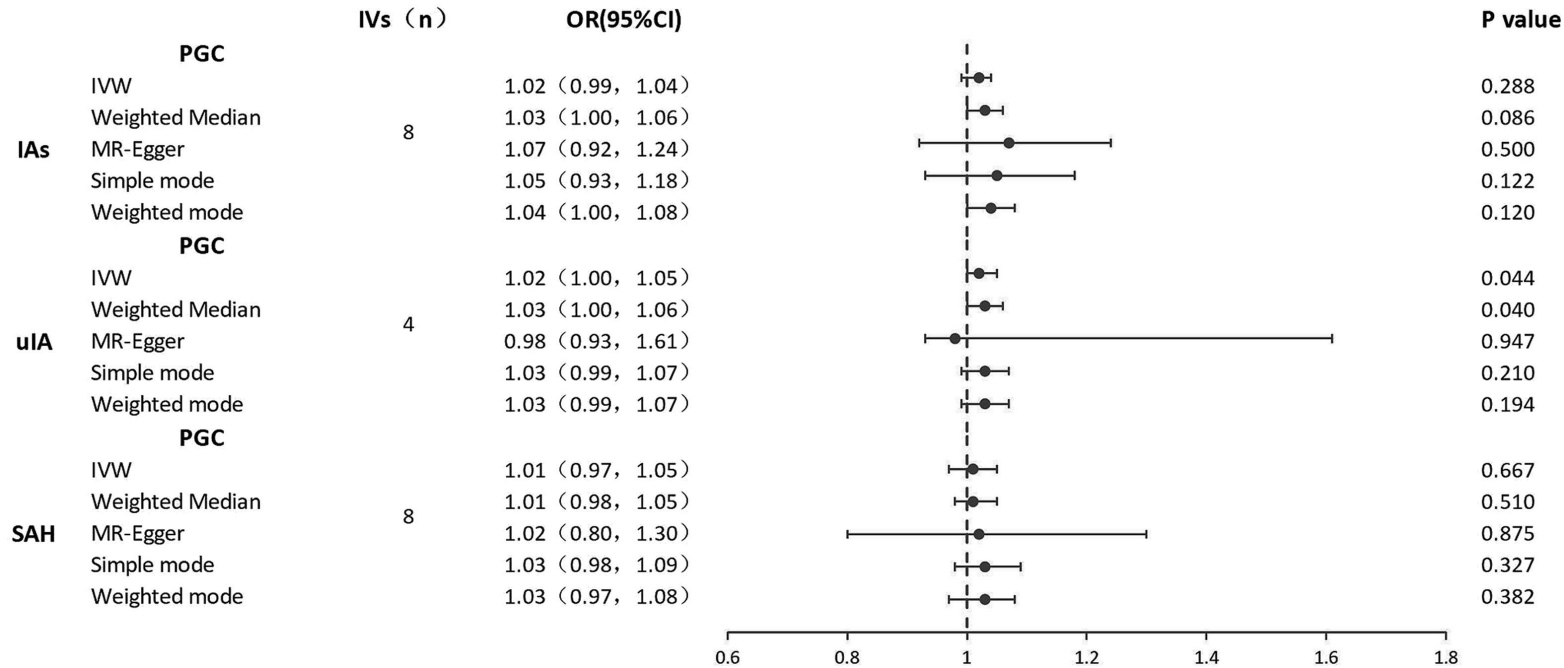
Forest plots of causal effects of IAs, uIA, and SAH on depression. IAs, intracranial aneurysms; uIA, unruptured intracranial aneurysm; SAH, subarachnoid hemorrhage; IVs, instrumental variables; IVW, inverse-variance weighted; MR, Mendelian randomization; OR, odds ratio; 95% CI, 95% confidence interval.

Results of the reverse MR analysis of the causal effect of IAs, SAH, and uIA on depression and the evaluation of pleiotropic effects are in Table 3. We also created scatter and forest plots for each pair of associations (Supplementary Figures S1D–F; Figure 3). MR results showed that only uIA was causally associated with depression in the IVW models (OR = 1.02, 95% CI 1.00–1.05, *p* = 0.044), which was positively associated with depression. No significant relations were found for IAs or SAH (IAs: OR = 1.01, 95% CI 0.99–1.04, *p* = 0.288; SAH: OR = 1.00, 95% CI 0.97–1.05, *p* = 0.667). Regarding the causal relations between uIA or SAH and depression, no horizontal pleiotropy was detected in the MR-Egger regression (uIA: egger_intercept = 0.0, *p* = 0.89; SAH: egger_intercept = 0.0, *p* = 0.92) or the MR-PRESSO global test (uIA: *p* = 0.43; SAH: *p* = 0.06). For the causal relation between IAs and depression, while MR-Egger regression suggested no horizontal pleiotropy (egger_intercept = 0.0, *p* = 0.64), MR-PRESSO found evidence of pleiotropy (*p* = 0.03). MR-PRESSO did not show any significant outliers of horizontal pleiotropy. Cochran’s *Q* and funnel plots (Supplementary Figures S2D–F) indicated no heterogeneities, except for the relation between SAH and depression risk. Leave-one-out analyses showed no significant SNPs aside from those for uIA (Supplementary Figures S3D–F).

**Table 3:** Effect estimates of associations between genetic instrumental variables for aneurysm and depression risk.

IAs	IVW		MR-Egger		MR PRESSO (outlier-corrected)		
	OR (95% CI)	Q (*p* value)	OR (95% CI)	Intercept (*p* value)	Outlier	OR (95% CI)	*p* for global test
Depression	1.01 (0.99, 1.04)	13.3 (0.06)	1.05 (0.93, 1.18)	0.0 (0.64)	0	NA	0.03

SAH	IVW		MR-Egger		MR PRESSO (outlier-corrected)		
	OR (95% CI)	Q (*p* value)	OR (95% CI)	Intercept (*p* value)	outlier	OR (95% CI)	*p* for global test
Depression	1.00 (0.97, 1.05)	11.3 (0.02)	1.02 (0.80, 1.30)	0.0 (0.92)	0	NA	0.06

uIA	IVW		MR-Egger		MR PRESSO (outlier-corrected)		
	OR (95% CI)	Q (*p* value)	OR (95% CI)	Intercept (*p* value)	Outlier	OR (95% CI)	*p* for global test
Depression	1.02 (1.00, 1.05)	3.5 (0.32)	0.98 (0.53, 1.81)	0.0 (0.89)	0	NA	0.43

IAs, intracranial aneurysms; uIA, unruptured intracranial aneurysm; SAH, subarachnoid hemorrhage; OR, odds ratio; 95% CI, 95% confidence interval; Q, Cochran’s Q; NA, not applicable.

## 4 Discussion

Herein, we assessed the causal relations between depression and IAs. Two-sample bidirectional MR analyses detected that genetic propensity for depression was positively associated with IAs, SAH, and uIA risks. Reverse MR analyses showed that only genetic link to uIA was associated with increased depression risk. No evidence was found to indicate a genetic link with either IAs or SAH in association with depression.

Observational studies have revealed bidirectional correlations between depression and IAs. Marijnissen et al. discovered that depression is a risk factor for IAs (11), consistent with our results. Individuals diagnosed with depression exhibit a 43% elevated risk of stroke and have an average two-point higher National Institutes of Health Stroke Scale score than do those without pre-stroke depression (26, 27). Pre-stroke depression is likely a substantial modifiable risk factor for post-stroke depression and functional impairment (28, 29). A meta-analysis of a cumulative ~700,000 participants by Barlinn et al. found that depression increases the risk of first-ever stroke by 40% in the general population (26). A further registry study reported evidence of an association between hospitalization for depression and subsequent stroke (30). Pharmacological interferences with platelet aggregation caused by antidepressant medication may increase stroke risk in those with depression (31). Hypothalamic–pituitary–adrenal (HPA) axis dysregulation related to stress and depression may increase circulating catecholamines, endothelial dysfunction, and platelet activation, resulting in a hypercoagulable condition and raising stroke risk (32, 33). Previous studies have also suggested that depression has unique underlying cerebral pathomechanisms, including cerebral inflammation, HPA axis dysregulation, increased platelet reactivity, and autonomic dysfunction (34). The causal relations between depression and IAs, uIA, and SAH herein thus reinforce the notion that depression prevention and early diagnosis may help prevent IAs,uIA, and SAH. The distinct clinical prognosis of uIA and SAH suggest that alleviating depression may reduce IAs rupture risk, particularly in patients with uIA in whom long-term observation or watchful waiting is preferred over surgical intervention. However, the exact mechanisms underlying the causal association between depression and IAs remains unclear. Subsequent studies should thus concentrate on the specific mechanisms mediating these associations, and on pharmacological treatments for depression.

Regarding the reverse direction, living with uIA without treatment may lower quality of life and lead to mental health issues like anxiety and depression (35, 36). Chinese patients with untreated uIA tend to suffer from short-term depression, anxiety, and reduced quality of life after diagnosis (37). These findings are consistent with our study results showing that uIA is positively related to depression risk. Patients diagnosed with uIA may develop depression from concerns about the uIA rupturing. In patients with uIA, rupture risk is the most apparent cause of preoperative anxiety and depression (37). Recent studies emphasize the significance of psychological factors and quality of life in uIA management strategies (38, 39). Our findings support the notion that attending to the occurrence of depression in patients with uIA is warranted. Furthermore, long-term depressive symptoms persist after SAH in 72% of patients (40). Depression after stroke is generally considered a chronic illness, with prevalence and incidence of ~30% and ~ 15%, respectively, for 1–15 years post-stroke (41). After SAH, up to one in three patients may develop pituitary dysfunction (42), which may facilitate development of depression (43). Patients with depression after SAH also have lower basal cortisol levels (44). Yet our MR analyses revealed no evidence of support for causal effects of IAs or SAH on depression, indicating that the previously observed associations may be due to confounds. Studies of the development of depression after SAH have found that comorbid cognitive impairment, fatigue, post-traumatic stress disorder, and physical disability increase depression risk (9).

This study had several major strengths. First, it is the first two-sample MR study to identify a causal effect between depression and IAs, allowed by genotype-based random distribution. Second, this design avoids the possible effects of reverse causation and potential confounding factors in conventional studies, allowing investigation of causal relations. Third, each exposure had an F-statistic >10, indicating an absence of weak instrument bias. Finally, we tested MR model assumptions through several primary sensitivity analyses.

Several limitations must also be acknowledged. First, our results were derived only from populations of European ancestry; it is therefore essential to exercise caution when applying them to non-European populations, as environment and ethnicity may influence these relations. Second, a relatively small number of strongly correlated SNPs were selected for MR analysis of reverse causality; invalid results may be due to deficient SNPs, which would limit our ability to identify genuine causal relations. Third, not all SNPs were examined; some may have been removed due to LD, potentially impacting the results. Finally, as is true of nearly all MR analyses, it is possible that the IV SNPs we used were related to unmeasured confounders.

## 5 Conclusion

These two-sample bi-directional MR analyses of depression and IAs, based on large-scale GWAS summary statistics, provide strong evidence of a causal association between depression and IAs, including IAs, SAH, and uIA. Reverse MR analyses support a causal effect of uIA on depression, though not of IAs or SAH on depression. These findings support the recommendation that depression prevention and treatment may mitigate IAs occurrence and progression, and that depression should be closely monitored among patients diagnosed with uIA.

## Data availability statement

The original contributions presented in the study are included in the article/Supplementary material, further inquiries can be directed to the corresponding author.

## Ethics statement

Ethical approval was not required for the study involving humans in accordance with the local legislation and institutional requirements. Written informed consent to participate in this study was not required from the participants or the participants’ legal guardians/next of kin in accordance with the national legislation and the institutional requirements.

## Author contributions

JW: Writing – original draft, Writing – review & editing. HS: Writing – original draft, Writing – review & editing. JM: Writing – review & editing.
